# Interpreting flow cytometry with caution: A case report of follicular lymphoma in leukemic phase misdiagnosed as chronic lymphocytic leukemia

**DOI:** 10.3389/fonc.2023.1170598

**Published:** 2023-03-27

**Authors:** Yuan Ren, Congwei Jia, Miao Chen, Wei Wang, Wei Zhang

**Affiliations:** ^1^ Department of Hematology, Peking Union Medical College Hospital, Chinese Academy of Medical Sciences & Peking Union Medical College, Beijing, China; ^2^ 4+4 Medical Doctor Program, Chinese Academy of Medical Sciences & Peking Union Medical College, Beijing, China; ^3^ Department of Pathology, Peking Union Medical College Hospital, Chinese Academy of Medical Sciences & Peking Union Medical College, Beijing, China

**Keywords:** chronic lymphocytic leukemia, misdiagnosis, immunophenotyping, follicular lymphoma, case report

## Abstract

Chronic lymphocytic leukemia (CLL) is a subtype of mature B-cell proliferative neoplasms characterized by abnormally increasing lymphocytes in circulation. The diagnosis of CLL is usually established on peripheral blood analysis and typical flow cytometric immunophenotype rather than biopsy. In particular, the high RMH (Royal Marsden Hospital Scoring System for CLL) score of immunophenotype has a highly sensitive weight for diagnostic value. However, immunophenotyping by flow cytometry may also be misleading in specific clinical situations. Here, we report a case on admission with lymphadenopathy and lymphocytosis, misdiagnosed as chronic lymphocytic leukemia by flow cytometry initially but finally confirmed as follicular lymphoma (FL) in the leukemic phase *via* lymph node biopsy. Since FL in the leukemic phase is uncommon at the time of diagnosis and indicates a poorer prognosis of FL, such misdiagnosis is worthy of attention. It is also thought-provoking that there had been conflicts between immunophenotype of bone marrow and immunohistochemistry of lymph node. Our case report aims to remind clinicians’ awareness that the immunophenotyping by flow cytometric analysis needs to be interpreted with caution especially when the results cannot account for all of clinical features, and it is significant to make the right decision about when to conduct further examination including lymph node biopsy for avoiding misdiagnosis.

## Introduction

Chronic lymphocytic leukemia (CLL) is a common type of indolent B-cell lymphoma that occurs mainly in middle-aged and elderly people, especially in Western countries. According to recent guidelines (1–3), peripheral blood routine, smear, and flow cytometric analysis are adequate for diagnosing CLL, and it is not generally required to conduct lymph node biopsy. Despite a high RMH (Royal Marsden Hospital Scoring System for CLL) score (≥4) of immunophenotyping representing high sensitivity in distinguishing CLL from other B-cell proliferative disorders (4), the results of immunophenotype by flow cytometry need to be interpreted with caution, particularly in need of combination with clinical features. Herein, we report a case of a young male patient misdiagnosed with CLL by flow cytometry initially but finally confirmed as follicular lymphoma *via* lymph node biopsy, in hopes of reminding clinicians’ awareness.

## Case presentation

**Figure 1:**
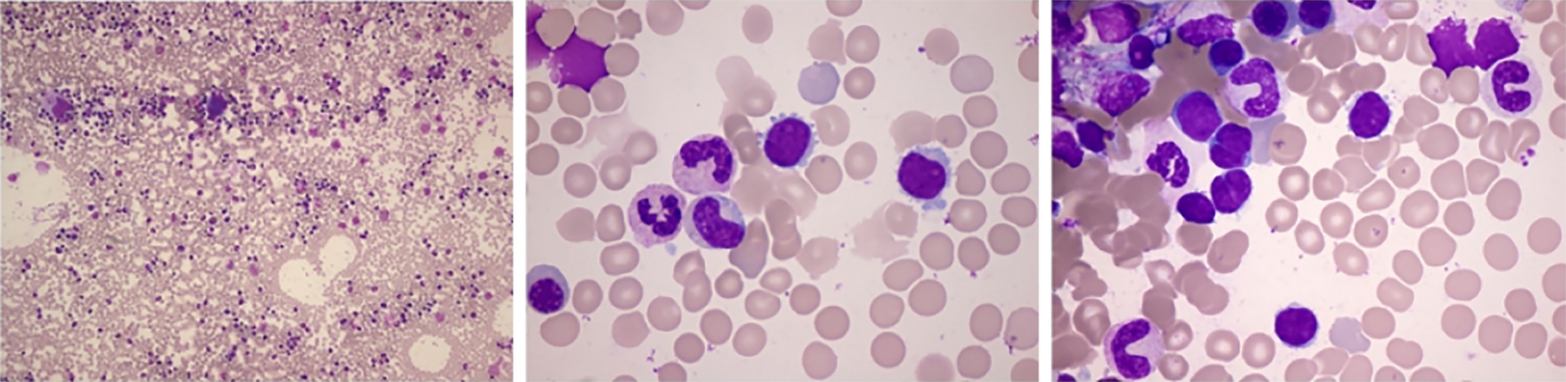
Bone marrow smear: active proliferation. The positive rate of periodic acid–Schiff staining was 75%. Some heteromorphic lymphocytes were small to moderate in size with mature aggregated chromatin, scant cytoplasm, and irregular nuclear contours. The smear suggested lymphoproliferative diseases.

**Figure 2:**
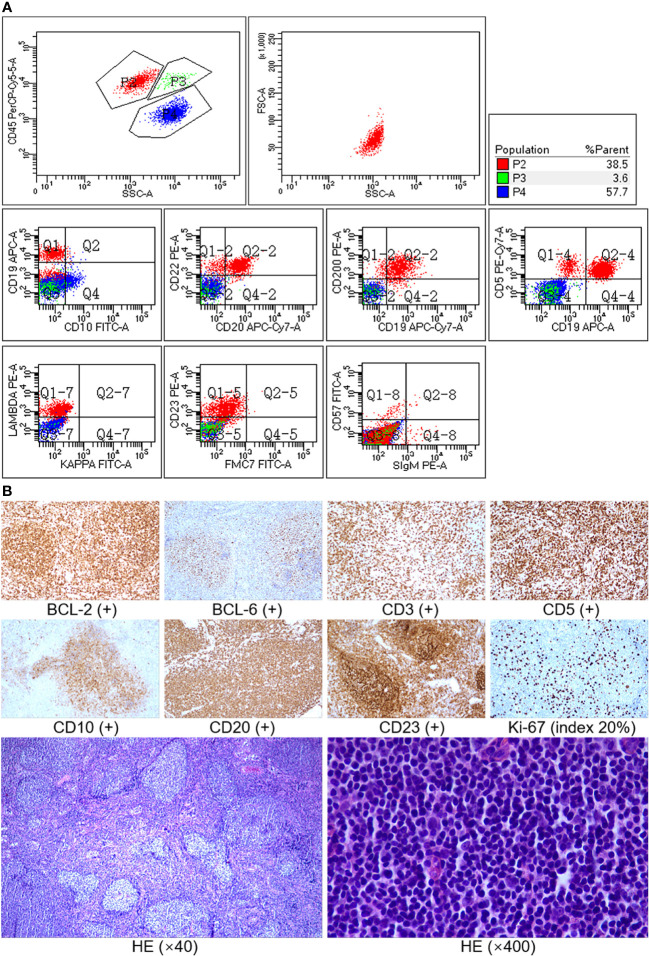
**(A)** Immunophenotype of bone marrow by flow cytometry. Method: sample staining (lyse and wash) by fluorescent-labeled monoclonal antibody was performed, a BD FACSCanto II flow cytometer was used, and the data were analyzed using BD FACSDiva software. Result: P2 (red) was lymphocytes, accounting for 38.5% of non-erythrocytes in the sample. B cells accounted for 85% of lymphocytes and mainly expressed CD19, CD20, CD22, CD5, CD23, and CD200 but did not express CD10, FMC7, and sIg (surface immunoglobulin), with lambda light-chain restriction. The immunophenotype of B cells was abnormal phenotype. **(B)** Immunohistochemistry and histopathology of lymph node biopsy. The neoplastic follicles were closely packed, showing an almost back-to-back pattern, and lacked mantle zones (low power, ×40). Most of the cells in the field were centrocytes (high power, ×400). Immunostaining showed that the tumor cells were positive for CD20, CD10, BCL-6, and BCL-2 and negative for CD3, CD5, and CD23, and the Ki-67 index was about 20%. CD3 and CD5 were positive for background T cells, and CD23 was positive for follicular dendritic cells, while follicular lymphoma cells did not express CD5 and CD23.

A 42-year-old man without any past medical history was admitted in our hospital with 1 year of weight loss and night sweats. Physical examination revealed lymphadenopathy and massively enlarged spleen (reached the level of umbilicus). Peripheral blood analysis found a lymphocyte count of 16.80 × 10^9^/l, and abnormal lymphocytes accounted for 59% of white blood cells, whereas the hemoglobin concentration (144 g/l) and platelet counts (131 × 10^9^/l) were within the normal range. Serum biochemical examination showed an elevated lactate dehydrogenase (LDH) level of 267 U/l (upper limit of normal, 250 U/l) and a β2-microglobulin (β2-MG) level of 6.6 mg/l (reference range, 0.7–1.8 mg/l). Bone marrow smear showed an increase in mature heteromorphic lymphocytes (**Figure 1**), suggesting a chronic lymphoproliferative disease. The immunophenotype of bone marrow by flow cytometric analysis (**Figure 2A**) was an abnormal B-cell phenotype: CD19^+^CD20^+^CD5^+^CD23^+^CD200^+^CD22^+^CD10^-^FMC7^-^sIg^-^. Given that the RMH score calculated is 4, chronic lymphocytic leukemia was firstly diagnosed.

**Figure 3:**
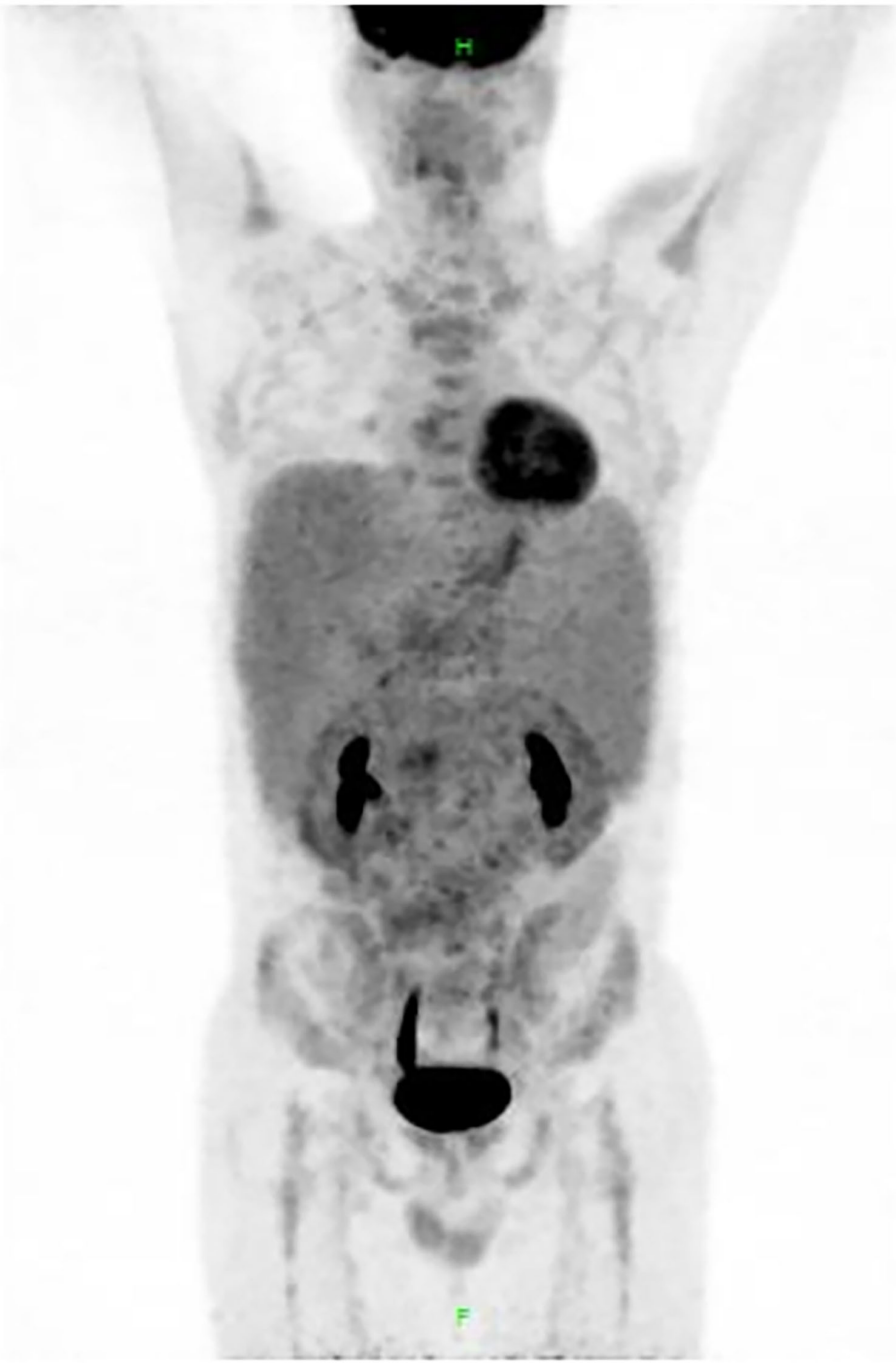
Results of positron emission tomography (PET)-CT. PET-CT scan revealed multiple lymphadenopathy (SUVmax 5.0) in the bilateral cervical, bilateral axillary, mediastinal, retroperitoneal, supra-mesenteric, iliac vessel, and bilateral inguinal region, and the maximum cross section of the lymph node mass was 10.9 cm × 10.2 cm. Liver (SUVmax 3.2) and spleen (SUVmax 3.0) were significantly enlarged. Metabolism of central and peripheral bone marrow was diffusely increased, with SUVmax of 3.6.

However, considering the patient’s young age and significantly enlarged spleen, we planned a further evaluation to confirm the diagnosis. PET-CT scan revealed multiple (cervical, axillary, inguinal, retroperitoneal, etc.) lymphadenopathy (maximum cross section: 10.9 cm × 10.2 cm) and obvious hepatosplenomegaly, with a maximum SUV of 5.0 (**Figure 3**). Biopsy of the left inguinal lymph node was scheduled (see pathological details in **Figure 2B**), and the patient was finally diagnosed with follicular lymphoma (FL) in the leukemic phase. Meanwhile, DNA sequencing detected CREBBP mutation, which also supports common gene features of FL (5). As per extranodal organs such as liver and bone marrow involved, the final Ann Arbor stage was IVB. FLIPI (Follicular Lymphoma International Prognostic Index)-1 and FLIPI-2 both scored 3 (high risk).

The treatment was initiated for ≥1 GELF criteria (any site >7 cm; B symptoms; splenomegaly; leukemic phase) that were met (6). Six cycles of the BR regimen (rituximab and bendamustine) were given, and the patient achieved complete remission (CR).

## Discussion

Reviewing this case, the young male patient mainly presented with B symptoms, lymphadenopathy, and palpable splenomegaly, and peripheral blood examination indicated a prominent lymphocytosis accompanied by several biochemical abnormalities. Next, when interpreting the results of immunophenotype, although the RMH score of 4 is highly sensitive for diagnosing CLL, we still cannot draw a conclusion directly based on flow cytometry since there existed some clinical features of the case mismatched with CLL. Here, the reasons that drive the clinician’s critical decision to lymph node biopsy are as follows: First, the age of the patient is relatively young compared to the median age of CLL (72-year-old) at diagnosis (1). Second, the patient had marked splenomegaly with elevated serum LDH and β2-MG levels, while his lymphocyte count was only 16.80 × 10^9^/l. Third, on PET-CT scanning CLL overall has a lower fluorine-18 fluoro-deoxyglucose avidity, namely, lower SUV value (mean: 2.5), compared with other indolent lymphomas such as FL (mean SUV: 7.7) (7). Due to the above reasons, further examination especially lymph node biopsy was essential.

In our report, of concern is that the initial immunophenotype (CD5^+^CD23^+^CD200^+^CD10^-^FMC7^-^sIg^-^) of bone marrow by flow cytometry did not coincide with immunohistochemistry (CD5^-^CD23^-^CD10^+^BCL-2^+^BCL-6^+^) of lymph node pathology. By reviewing a previous study of FL in the leukemic phase, we found a similar discordance. As Maeshima et al. mentioned (8), the incidence of CD10 positivity was lower in the bone marrow compared to the primary site (lymph node); even the histological grades of FL were discordant between the bone marrow and other sites. Dogan et al. illustrated (9) that because of a lack of follicle germinal center activation markers and a low proliferation fraction, diffusely infiltrating lymphoma cells in the bone marrow may be negative for CD10 expression. Regarding why the bone marrow immunophenotyping showed CLL-like characteristics in our case, we assume that the cell surface immunophenotype had been transformed when follicular lymphoma cells involved bone marrow and peripheral blood. It probably related to the unique hematopoietic microenvironment of bone marrow, resulting in changes of cell growth patterns. Such unusual presentation of the leukemic FL is not completely observed or understood. There remain several unanswered questions about the mechanism behind this phenomenon to be explored.

Additionally in previous retrospective cohort studies, the leukemic phase of FL patients is rare and associated with shorter PFS independently of the FLIPI score (10–12). Therefore, it is fairly significant that follicular lymphoma in the leukemic phase is timely and correctly diagnosed.

## Conclusion

For patients with a CLL-like immunophenotype (RMH score ≥4) while clinical features are not completely consistent with CLL, the conclusion drawn from flow cytometry should be interpreted cautiously. As reported in our case, manifestations of FL in the leukemic phase in some ways are mimicking those of CLL, thus requiring careful differential diagnosis. Even if small lymphoma cells in the bone marrow resemble the CLL immunophenotype and are negative for CD10, FL cannot be excluded. In summary, the final diagnosis must not be made only on the basis of the immunophenotypic data but must be a synthesis that the clinician makes considering all the clinical and laboratory aspects. To avoid misdiagnosis, lymph node biopsy should be performed positively in the presence of atypical clinical findings.

## Data availability statement

The original contributions presented in the study are included in the article/supplementary material. Further inquiries can be directed to the corresponding author.

## Ethics statement

Written informed consent was obtained from the individual(s) for the publication of any potentially identifiable images or data included in this article.

## Author contributions

YR wrote the first draft of the manuscript, CJ, MC and WZ provided the case data, and WW revised the final draft. All authors contributed to the article and approved the submitted version.
