# Corrigendum: Interpreting flow cytometry with caution: a case report of follicular lymphoma in leukemic phase misdiagnosed as chronic lymphocytic leukemia

**DOI:** 10.3389/fonc.2023.1229649

**Published:** 2023-06-06

**Authors:** Yuan Ren, Congwei Jia, Miao Chen, Wei Wang, Wei Zhang

**Affiliations:** ^1^ Department of Hematology, Peking Union Medical College Hospital, Chinese Academy of Medical Sciences & Peking Union Medical College, Beijing, China; ^2^ 4+4 Medical Doctor Program, Chinese Academy of Medical Sciences & Peking Union Medical College, Beijing, China; ^3^ Department of Pathology, Peking Union Medical College Hospital, Chinese Academy of Medical Sciences & Peking Union Medical College, Beijing, China

**Keywords:** chronic lymphocytic leukemia, misdiagnosis, immunophenotyping, follicular lymphoma, case report

In the published article, there was an error in the Funding statement. The funding statement was forgotten. The correct Funding statement appears below.

**FUNDING**

This work was supported by grants from the National High Level Hospital Clinical Research Funding of China (No. 2022-PUMCH-A-194).

The authors apologize for this error and state that this does not change the scientific conclusions of the article in any way. The original article has been updated.

